# Supplementary figures and images for: Seizure occurrence in FCD type II is predicted by lesion position and linked to cytoarchitectural alterations
Source: Acta Neuropathol Commun. 2025 Dec 9;13:251. doi: 10.1186/s40478-025-02166-x (PMC12690940; doi:10.1186/s40478-025-02166-x)

**A**

Seizures/Day

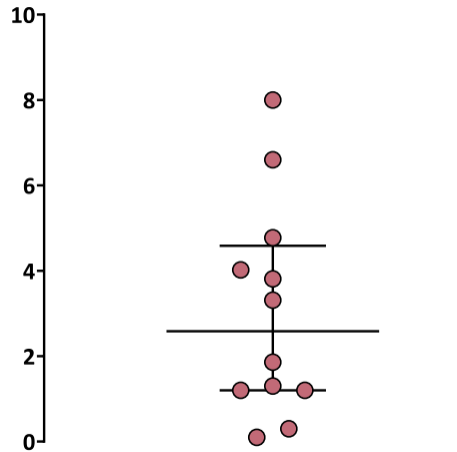**B**

Mean Seizure Duration (s)

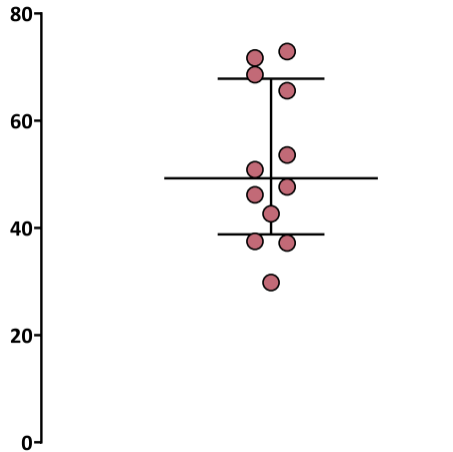**C**

IED rate/min

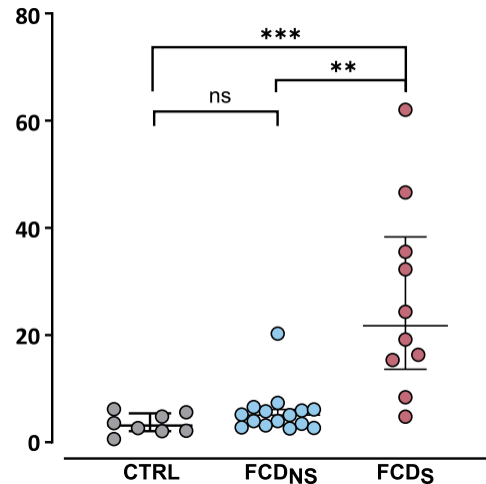

Supplement: Supplementary file 1 — Supplementary Material 1. Fig. 1 Animals with seizures involved in the study. (A) Number of seizures per day and their mean duration (B) for all sizing mice included in this project; points denote individual mice, middle lines = medians, whiskers = IQRs. (C) Comparison of interictal epileptiform discharge (IED) frequency between control animals and FCD mice without or with seizures. [file 40478_2025_2166_MOESM1_ESM.pdf]

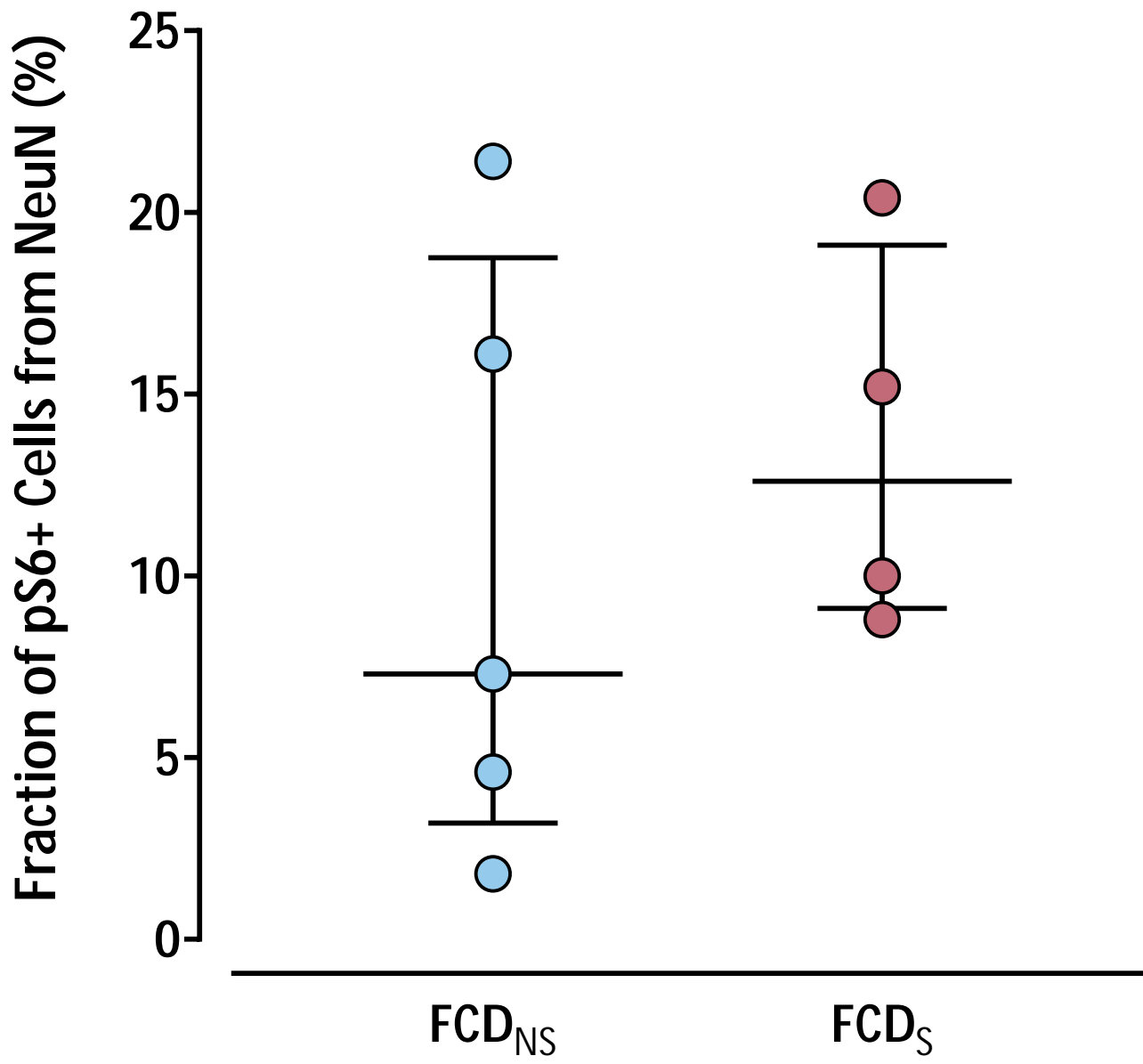

Supplement: Supplementary file 3 — Supplementary Material 3. Fig. 3 Fraction of mTOR-hyperactivated neurons in FCD in seizing and non-seizing animals. Points denote individual mice, middle lines = medians, whiskers = IQRs. [file 40478_2025_2166_MOESM3_ESM.pdf]

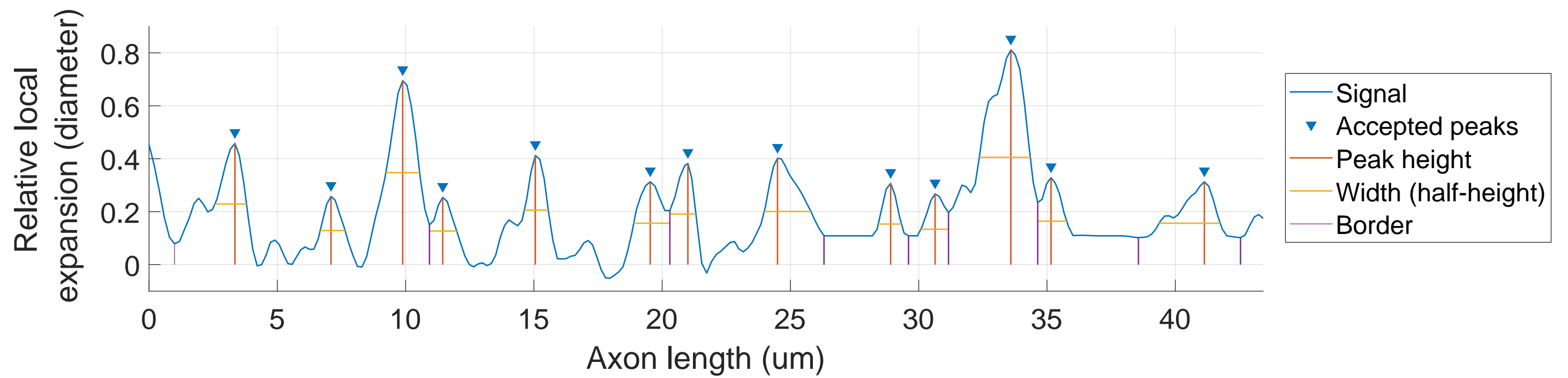

Supplement: Supplementary file 4 — Supplementary Material 4. Fig. 4 Axonal boutons detection parameters. Individual parameters defined in Methods. [file 40478_2025_2166_MOESM4_ESM.pdf]

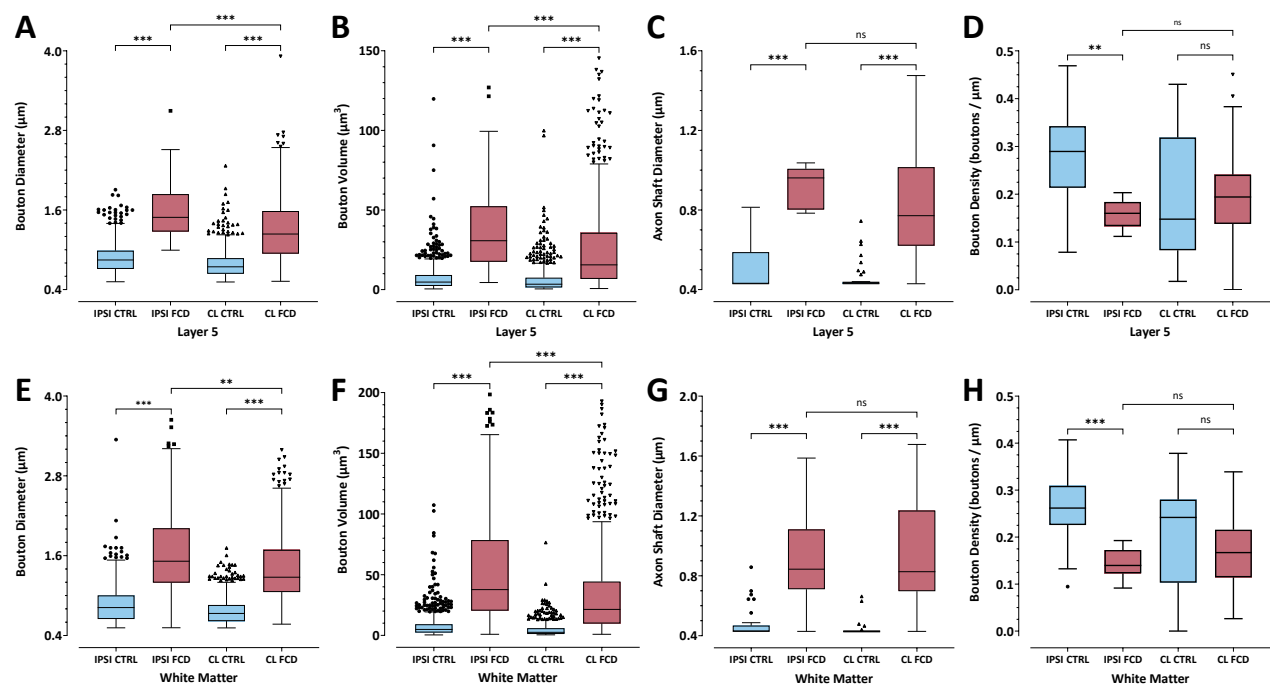

Supplement: Supplementary file 5 — Supplementary Material 5. Fig. 5 Parameters of ipsilateral and contralateral axonal boutons in layer 5 and white matter. (A, E) Tukey boxplots of axonal bouton diameters. (B, F) Tukey boxplots of axonal bouton volumes. (C, G) Tukey boxplots of axon shaft diameters. (D, H) Tukey boxplots of axonal bouton density. (A–H) Measurements taken from either layer 5 or the white matter in either control animals (CTRL) or animals with lesions (FCD), both on the ipsilateral (IPSI) and homotopic contralateral (CL) areas. [file 40478_2025_2166_MOESM5_ESM.pdf]

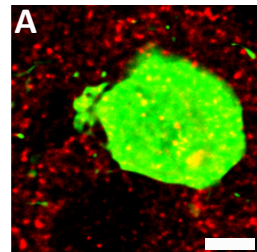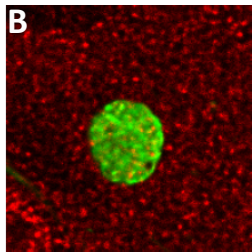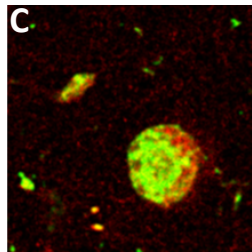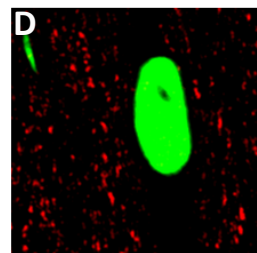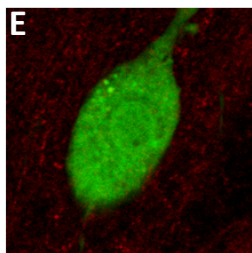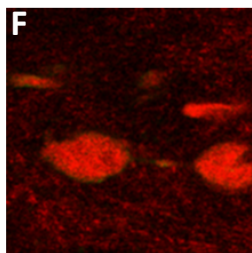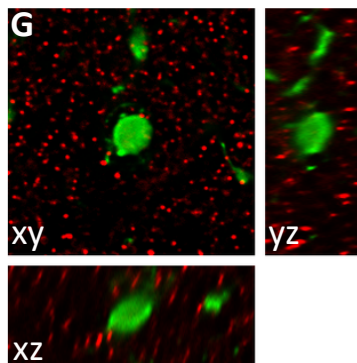

Supplement: Supplementary file 6 — Supplementary Material 6. Fig. 6 Examples of axonal varicosities in the contralateral cortex and the corpus callosum stained for synapse-related proteins. Giant axonal boutons of dysmorphic neurons were visualized in the contralateral (CL) cortex (A–C, G) and in the corpus callosum (CC) (D–F). (A) vGlut1 puncta (red) colocalized with axonal boutons (green) in the CL cortex, but not in the CC (D). (B) Synapsin puncta (red) were present within boutons (green) in the CL cortex, but absent from CC boutons (E). (C) Synaptic terminals of dysmorphic neurons expressing tdTomato and the synaptophysin–EGFP fusion protein. Clusters of synaptophysin–EGFP (green) were evident in boutons in the CL cortex, but not in axonal varicosities in the CC (F). (G) An axonal bouton of an EGFP-expressing dysmorphic neuron labeled against PSD95 (red). [file 40478_2025_2166_MOESM6_ESM.pdf]
