# Supplementary material for: Seizure occurrence in FCD type II is predicted by lesion position and linked to cytoarchitectural alterations
Source: Acta Neuropathol Commun. 2025 Dec 9;13:251. doi: 10.1186/s40478-025-02166-x (PMC12690940; doi:10.1186/s40478-025-02166-x)
Supplement: Supplementary file 2 — Supplementary Material 2. Fig. 2 Background-insensitive classification algorithm of pS6-positivity in IHC datasets. [file 40478_2025_2166_MOESM2_ESM.pdf]

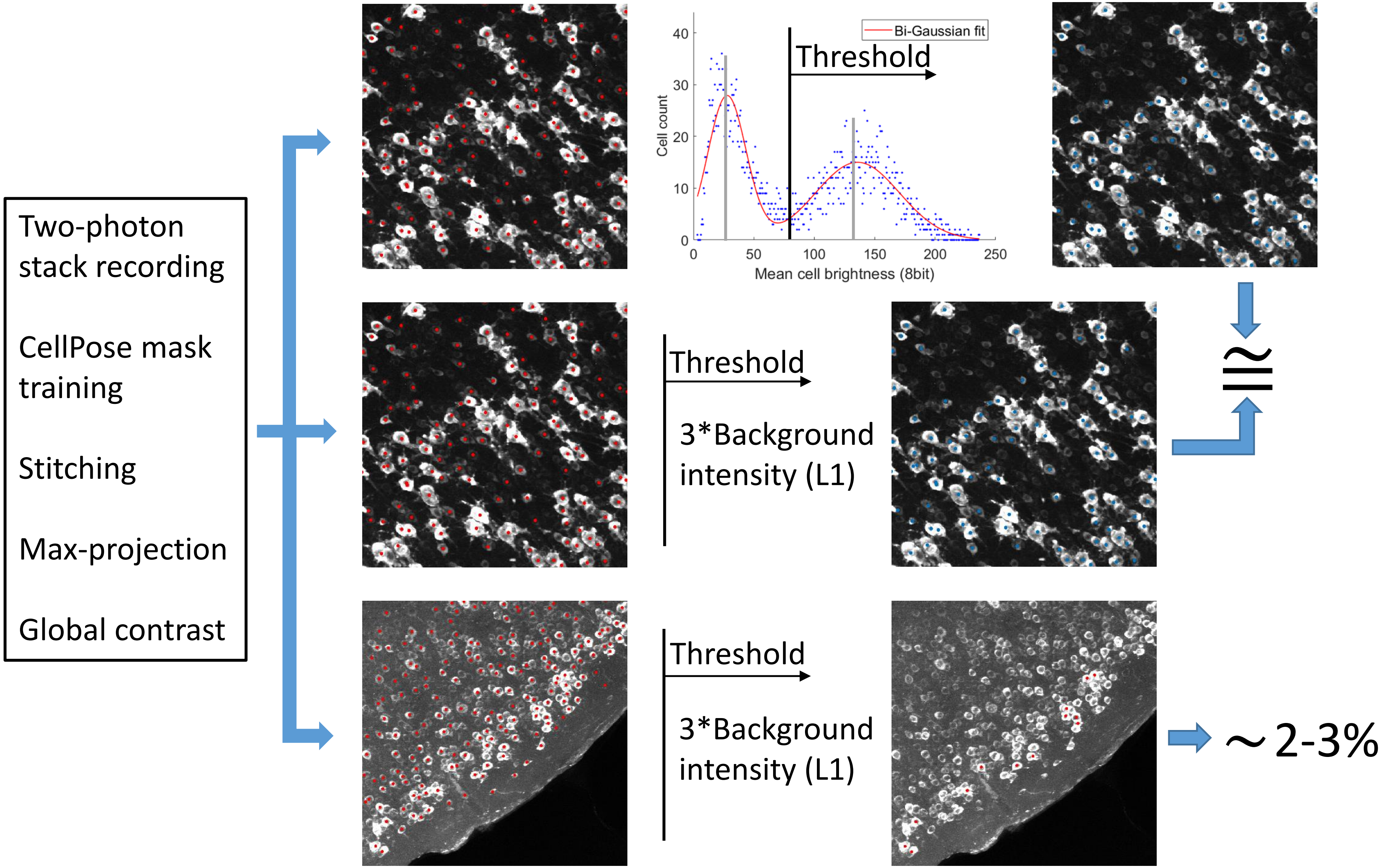

Two-photon stack recording
CellPose mask training
Stitching
Max-projection
Global contrast
Bi-Gaussian fit
Threshold
Cell count
Mean cell brightness (8bit)
0
50
100
150
200
250
10
20
30
40
Threshold
3*Background intensity (L1)
Threshold
3*Background intensity (L1)
≅
~2-3%
