# Supplementary material for: Seizure occurrence in FCD type II is predicted by lesion position and linked to cytoarchitectural alterations
Source: Acta Neuropathol Commun. 2025 Dec 9;13:251. doi: 10.1186/s40478-025-02166-x (PMC12690940; doi:10.1186/s40478-025-02166-x)
Supplement: Supplementary file 7 — Supplementary Material 7. Table 1. Complete values to Supplementary Fig. 5. [file 40478_2025_2166_MOESM7_ESM.docx]

**Supplementary Tab. 1 - Complete values to Supplementary Fig. 5**

Legend: **IC** = ipsilateral control; **IF** = ipsilateral FCD; **CC** = contralateral control; **CF** = contralateral FCD; **IQR** = interquartile interval; **N** = number of boutons/shafts, mult. **p** = p values from multiple comparisons tests.

| **Sup. 5 Subplot** | **Median** | | | | **IQR** | | | | **N** | | | | ***P*** | ***mult. p*** (ICvIF, CCvCF, IFvCF) |
| --- | --- | --- | --- | --- | --- | --- | --- | --- | --- | --- | --- | --- | --- | --- |
|  | **IC** | **IF** | **CC** | **CF** | **IC** | **IF** | **CC** | **CF** | **IC** | **IF** | **CC** | **CF** |  |  |
| **A** (**μm)** | 0.85 | 1.5 | 0.74 | 1.2 | 0.71–0.99 | 1.3–1.8 | 0.64–0.87 | 0.94–1.6 | 1048 | 115 | 666 | 684 | <0.001 | <0.001 ALL |
| **B** (**μm^3^)** | 4.7 | 31 | 3.4 | 16 | 2.3–9.2 | 17–52 | 1.4–7.5 | 6.7–36 | 1048 | 115 | 666 | 684 | <0.001 | <0.001 ALL |
| **C** (**μm)** | 0.43 | 0.96 | 0.43 | 0.77 | 0.43–0.59 | 0.80–1.0 | 0.43–0.44 | 0.62–1.0 | 46 | 9 | 36 | 54 | <0.001 | <0.001, <0.001, 0.61 |
| **D** (**μm^-1^)** | 0.29 | 0.16 | 0.15 | 0.19 | 0.21–0.34 | 0.13–0.18 | 0.082–0.32 | 0.14–0.24 | 46 | 9 | 36 | 54 | <0.001 | 0.002, >0.99, 0.52 |
| **E** (**μm)** | 0.82 | 1.5 | 0.73 | 1.28 | 0.65–1.0 | 1.2–2.0 | 0.62–0.86 | 1.1–1.7 | 966 | 237 | 716 | 632 | <0.001 | <0.001, <0.001, 0.004 |
| **F** (**μm^3^)** | 4.9 | 38 | 2.5 | 21 | 2.3–9.2 | 20–78 | 1.2–5.9 | 9.8–44 | 966 | 237 | 716 | 632 | <0.001 | <0.001 ALL |
| **G** (**μm)** | 0.43 | 0.84 | 0.43 | 0.83 | 0.43–0.47 | 0.71–1.1 | 0.43–0.43 | 0.70–1.2 | 46 | 19 | 38 | 45 | <0.001 | <0.001, <0.001, >0.99 |
| **H** (**μm^-1^)** | 0.26 | 0.14 | 0.24 | 0.17 | 0.23–0.31 | 0.12–0.17 | 0.10–0.28 | 0.11–0.22 | 46 | 19 | 38 | 45 | <0.001 | <0.001, 0.09, 0.90 |
